# Analysis of Relationships between *DAT1* Polymorphism Variants, Personality Dimensions, and Anxiety in New Psychoactive Substance (Designer Drug) (NPS) Users

**DOI:** 10.3390/genes12121977

**Published:** 2021-12-10

**Authors:** Jolanta Chmielowiec, Krzysztof Chmielowiec, Jolanta Masiak, Tomasz Pawłowski, Dariusz Larysz, Anna Grzywacz

**Affiliations:** 1 Department of Hygiene and Epidemiology, Collegium Medicum, University of Zielona Góra, 28 Zyty St., 65-046 Zielona Góra, Poland; chmiele1@o2.pl (J.C.); chmiele@vp.pl (K.C.); 2 Second Department of Psychiatry and Psychiatric Rehabilitation, Medical University of Lublin, Głuska St., 20-059 Lublin, Poland; jolanta.masiak@umlub.pl; 3 Division of Psychotherapy and Psychosomatic Medicine, Wroclaw Medical University, Wyb. L. Pasteura 10 St., 50-367 Wroclaw, Poland; tomasz.pawlowski@umw.edu.pl; 4 109 Military Hospital with Cutpatient Cinic in Szczecin, Piotra Skargi 9-11 St., 70-965 Szczecin, Poland; dariuszlarysz@hotmail.com; 5 Independent Laboratory of Health Promotion, Pomeranian Medical University in Szczecin, 11 Chlapowskiego St., 70-204 Szczecin, Poland

**Keywords:** addiction, dependence, dopamine, personality traits, genetics

## Abstract

The use of ‘new psychoactive substances’ appears to be increasingly common. The aim of this study was to examine biological and personality determinants in individuals who choose to use these substances, which may help in the prevention and treatment of psychoactive substance use disorders. The study group consisted of 374 male volunteers; all were users of ‘new psychoactive substances’ (NPS). The NPS users were recruited after they had abstained—for at least 3 months—from any substance of abuse in addiction treatment facilities. The NPS patients and the control subjects were examined by a psychiatrist using the Mini-International Neuropsychiatric Interview (M.I.N.I.), the NEO Five-Factor Personality Inventory (NEO-FFI), and the State-Trait Anxiety Inventory (STAI) scales. The real-time PCR method was used for genotyping. When we compared the controls with the study group, statistically significant interactions were found between *DAT1* polymorphism, neuroticism, and NPS use. NPS use and *DAT1* polymorphism were associated with a higher level of neuroticism on the NEO-FFI scale. The study group of NPS users showed a higher severity of anxiety symptoms, both in terms of trait and state, compared to the control group. The results may support the idea that neuroticism and anxiety correlate strongly with coping motives for using NPS.

## 1. Introduction

The term ‘new psychoactive substance’ was defined by the European Union as a new narcotic or psychotropic drug, in pure form or in a preparation, that is not scheduled under the Single Convention on Narcotic Drugs of 1961 or the Convention on Psychotropic Substances of 1971, but which may pose a public health threat comparable to that posed by substances listed in those conventions (Council of the European Union decision 2005/387/JHA) [1].

The U.S. Drug Enforcement Administration (DEA) recognizes seven types of de-signer drugs: cannabinoids, phenethylamines, phencyclidines or arylcyclohexamines, tryptamines, piperazines, pipradrols, N-ring systems [2]. The speed at which new psychoactive substances have been introduced to the market has stabilized in recent years.

However, each year, the EU Early Warning System detects more than 50 new psychoactive substances, which increases the pool of previously reported new psychoactive substances. These substances resemble long-known psychoactive substances used without medical prescription, but they are not controlled under international drug laws. At the end of 2019, the EMCDDA (European Monitoring Centre for Drugs and Drug Addiction) monitored over 790 new psychoactive substances, 53 of which were first reported in Europe in 2019.

The most common types of new psychoactive substances in Europe are synthetic cannabinoids and cathinones (77% of all types reported in 2018). Synthetic drug use in Europe appears to be increasingly common, and new patterns of consumption present similar features in each European country. These patterns of consumption appear to have established across the European Union. New psychoactive substances are also used as a tool for managing chronic pain [3,4], as well as in social isolation associated with the COVID-19 pandemic [5,6].

In the existing research on personality and addictions, one of the most widely used personality models is the Big Five—a dimensional personality model. This model is based on trait-related personality conceptualizations and encompasses the following personality domains: extraversion, neuroticism, openness to new experiences, agreeableness, and conscientiousness [7]. Although it has been sufficiently confirmed by numerous research studies that personality traits influence the development, duration, and prognosis of substance use disorders [8], the data on associations between personality dimensions and the use of new psychoactive substances are limited.

A study conducted by Cohen et al. showed that NPS users exhibited a higher level of neuroticism and a lower level of agreeableness and extraversion than cannabis users and non-users. NPS users had lower scores for conscientiousness than natural cannabis users and non-users [9].

A study of polydrug users showed a low level of conscientiousness and a low level of agreeableness. High trait impulsivity and poor self-control were reported to constitute an additional risk to NPS and polydrug use severity and distinguished those seeking treatment from those who use NPS recreationally [10]. Polydrug users and alcohol users showed a high level of neuroticism and a low level of openness to new experiences in both groups, compared to healthy controls [11]. Studies of addiction confirmed higher levels of neuroticism in the examined individuals who had a diagnosis of substance and behavioral addictions [12,13,14,15,16], and severe neuroticism was reported to be associated with ‘self-medication’ of unpleasant or negative emotional states [17]. Mensen et al. [18] conducted a study in which synthetic cannabinoid users were compared to natural cannabis users; synthetic cannabinoid users had higher scores for anxiety and phobic anxiety. Studies analyzing gene variants examined in NPS patients are very limited. Pehlivan et al. [19] examined variants of the *COMT* (rs4680), *CNR2* (rs2501432), *CNR2* (rs2229579), *UCP2* (rs659366), and *IL-17* (rs763780) genes in patients with synthetic cannabinoid use disorder: the *CNR2* (rs2229579) and the *UCP2* (rs659366) variants were associated with synthetic cannabinoid use disorder. Pehlivan et al. [20] examined the relationship of global methylation with cannabinoid use disorder or synthetic cannabinoid use disorder, as well as methylation of the *NR3C1* gene promotor and the *NR3C1* BclI polymorphism. The existence of previous polysubstance abuse significantly influenced genotype distribution between the two groups, and global DNA methylation may have been associated with synthetic cannabinoid use disorder. Oyaci et al. [21] analyzed the association between cannabinoid use disorder/synthetic cannabinoid use disorder and methylation of the MB-COMT (membrane-bound catechol-O-methyltransferase) promotor or the *DRD2* gene in terms of gene variants; MB-COMT promoter methylation is believed to be associated with CUD/SCUD (cannabinoid use disorder/synthetic cannabinoid use disorder), but methylation of the *DRD2* gene is not. A common feature of addictive substances is their capacity to reinforce behavior and act as a powerful reward. There are numerous studies which have confirmed the critical role of DA in the rewarding effects of drugs of abuse [22,23,24]. The dopamine transporter (DAT) is a 12-membrane domain Na+/Cl−-dependent transport protein. It has a major role in dopaminergic neurotransmission, mediates the active reuptake of dopamine into the presynaptic neuron [25], and therefore regulates synaptic dopamine concentrations and the continuity of dopaminergic activity [26]. DAT may be involved in the pathophysiology of substance use disorder. The dopamine transporter gene (*DAT1*/gene symbol: SLC6A3), located on chromosome 5p15.3, encloses 15 exons ranging approximately 53 kb. As DAT is coded by a single-copy gene (SLC6A3), with its expression mostly restricted to the dopaminergic system, it points to a limited ability to be compensated for by other gene products. Polymorphisms in this gene may have a potentially large impact [27]. A variable number tandem repeat (VNTR) sequence in the 3′ untranslated region is considered to have a functional polymorphism regulating expression of the *DAT1* gene [28]. The *DAT1* gene consists of a polymorphic 40-base pair (bp) variable number tandem repeat (VNTR) in an untranslated region. Variants with 3 to 13 repeats of the 40 bp sequence have been identified, but the most often found are variants with 9 or 10 repeats, which are associated with high and low dopamine availability. Studies examining associations between *DAT1* polymorphisms and personality traits, as measured by the TCI, by Kim et al. in a Korean population found no associations in healthy Americans [29,30]. There are reports of an association between *DAT1* VNTR and novelty seeking, but they contradict each other [31,32]. Van Gestel et al. showed that in a Belgian cohort, particularly in females, novelty seeking was associated with *DAT1* polymorphisms [33].

The main aim of this study was to investigate associations between *DAT1* polymorphism variants, personality dimensions, and anxiety in users of new psychoactive substances (designer drugs) (NPS).

## 2. Materials and Methods

### 2.1. Subjects

The study group consisted of 374 male volunteers: users of ‘new psychoactive substances’ (NPS) (*n* = 73; mean age = 25.67, SD = 6.18, minimum = 17.00, maximum = 50.00), and healthy controls (*n* = 301; mean age = 22.13, SD = 4.57, minimum = 17.00, maximum = 50.00). Approval for the study was obtained from the Bioethics Committee of the Pomeranian Medical University in Szczecin (KB-0012/106/16). Informed written consent was received from the participants of the study. The study was conducted in the Independent Laboratory of Health Promotion. The users of ‘new psychoactive substances’ (NPS) were recruited after they had abstained for at least 3 months from any substance of abuse in addiction treatment facilities. The ‘new psychoactive substance’ (NPS) patients and the control subjects were examined by a psychiatrist using the Mini-International Neuropsychiatric Interview (M.I.N.I.), the NEO Five-Factor Personality Inventory (NEO-FFI), and the State-Trait Anxiety Inventory (STAI) scales.

The STAI is a questionnaire used to assess anxiety as a trait (A-Trait), which may be described as a permanent and enduring disposition to experience worries, stress, and discomfort, or as a state (A-state), including discomfort, fear, and the arousal of the autonomic nervous system occurring temporarily in relation to a particular situation. The NEO Personality Inventory (NEO Five-Factor Inventory, NEO-FFI) includes six dimensions for each of the five traits—extraversion (positive emotion, warmth, gregariousness, activity, excitement seeking, assertiveness), agreeableness (tender-mindedness, trust, altruism, straightforwardness, compliance, modesty), openness to experience (fantasy, feelings, esthetics, actions, values, ideas), conscientiousness (deliberation, competence, dutifulness, order, achievement striving, self-discipline), neuroticism (anxiety, vulnerability to stress, hostility, self-consciousness, impulsiveness, depression) [34].

The results of the NEO-FFI and STAI inventories were given as sten scores. Conversion of the raw score into the sten scale was performed according to the Polish norms. For adults, it was assumed that sten values of 1–2 were very low scores; values of 3–4 were low scores; values of 5–6 were average scores; values of 7–8 were high scores; and values of 9–10 were very high scores.

### 2.2. Genotyping

Tubes with EDTA (anticoagulant) were used to collect blood for the genetic assays. Genomic DNA from blood leukocytes was obtained using the High Pure Polymerase Chain Reaction (PCR) Template Preparation extraction kit (Roche Diagnostics, Mannheim, Germany). The process of extraction was conducted in accordance with the manufacturer’s instructions. The samples of DNA extracted in this way were stored at 4 °C until further analysis was performed.

Venous blood collected according to standard procedures was the source of genomic DNA. The PCR method was used to genotype the samples. The *DAT1* genotypes were grouped according to the presence of 9- or 10-repeat variants. Genotyping was performed using the PCR-VNTR method with primers: F: 50-TGT GGT GTA GGG AAC GGC CTG Ag 30, R: 50-CTT CCT GGA GGT CAC GGC TCA AGG 30, in a final volume of 25 L PCR mix per reaction, with l00 ng genomic DNA, 10 pmol of primers, 50 mM KCL, 10 mM TrisHCl, 1.5 mM MgCl_2_, 200 M dATP, dCTP, dTTP, and dGTP, and 0.8 U of the Tag polymerase. Conditions for the reaction: 5 min of initial denaturation at 94 °C; cycling: 55 s of denaturation at 94 °C, 50 s of primer hybridization at 55 °C, and 1 min of elongation at 72 °C, repeated in 30 cycles, with 10 min of final elongation at 72 °C. The amplified products were visualized using ethidium bromide-stained gel electrophoresis (3% agarose) and UV photography. The product lengths were 450 bp for 10-repeat alleles and 410 bp for 9-repeat alleles.

### 2.3. Statistical Analysis

Concordance between the genotype frequency distribution and Hardy–Weinberg equilibrium (HWE) was tested using the HWE software (http://www.oege.org/software/hwe-mr-calc.html, accessed on 20 March 2021). The relationships between *DAT1* variants, users of ‘new psychoactive substances’ (NPS), the control subjects, and the NEO Five-Factor Inventory (NEO-FFI) scores were analyzed using a multivariate analysis of factor effects ANOVA (NEO-FFI/scale STAI/× genetic feature × control and ‘new psychoactive substances’ (NPS) × (genetic feature × control and NPS)). The condition of homogeneity of variance was satisfied (Levene test *p* > 0.05). The variables did not present a normal distribution. The NEO Five-Factor Inventory scores (neuroticism, extraversion, openness, agreeableness, conscientiousness) were measured and compared using the Mann–Whitney U test. The *DAT1* genotype frequencies between healthy control subjects and users of ‘new psychoactive substances’ (NPS) were tested using the chi-square test. All computations were performed using STATISTICA 13 (Tibco Software Inc, Palo Alto, CA, USA) for Windows (Microsoft Corporation, Redmond, WA, USA).

## 3. Results

These frequency distributions accorded with the HWE both for the NPS users and control subjects (Table 1).

*DAT1* genotype frequencies in the studied sample did not differ between NPS users and control subjects (Table 2).

Compared to the control group, no statistically significant difference in the frequency of the genotype variants for the *DAT1* gene was found in the subjects using NPS (9/9 0.03 vs. 9/9 0.06, 9/10 0.33 vs. 9/10 0.39, 10/10 0.65 vs. 10/10 0.56, χ^2^ = 2.48, *p* = 0.289), and no statistically significant difference in the frequency for the *DAT1* alleles was found between the NPS users and the control group (9 0.19 vs. 9 0.25, 10 0.81 vs. 10 0.75, χ^2^ = 2.37, *p* = 0.124).

Compared to the controls, the study group subjects were observed to have significantly higher scores (Table 3) on the STAI state scale (M 6.08 vs. M 4.69, *p* < 0.001), the STAI trait scale (M 7.34 vs. M 5.16, *p* < 0.001), and the NEO Five-Factor Inventory neuroticism scale (M 6.92 vs. M 4.67, *p* < 0.001).

Compared to the controls, the study group had significantly lower scores (Table 3) on the NEO Five-Factor Inventory extraversion scale (M 5.78 vs. M 6.37, *p* = 0.0345) and the NEO Five-Factor Inventory agreeableness scale (M 4.67 vs. M 5.60, *p* = 0.0005).

**Figure 1:**
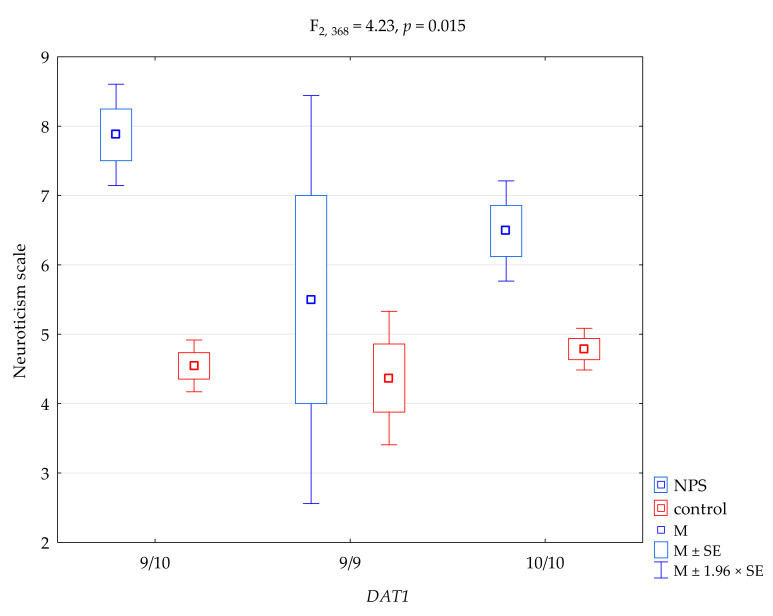
Interaction between users of ‘new psychoactive substances’ (NPS)/controls, *DAT1* polymorphisms, and the NEO-FFI neuroticism scale. M, mean; SE, standard error.

**Figure 2:**
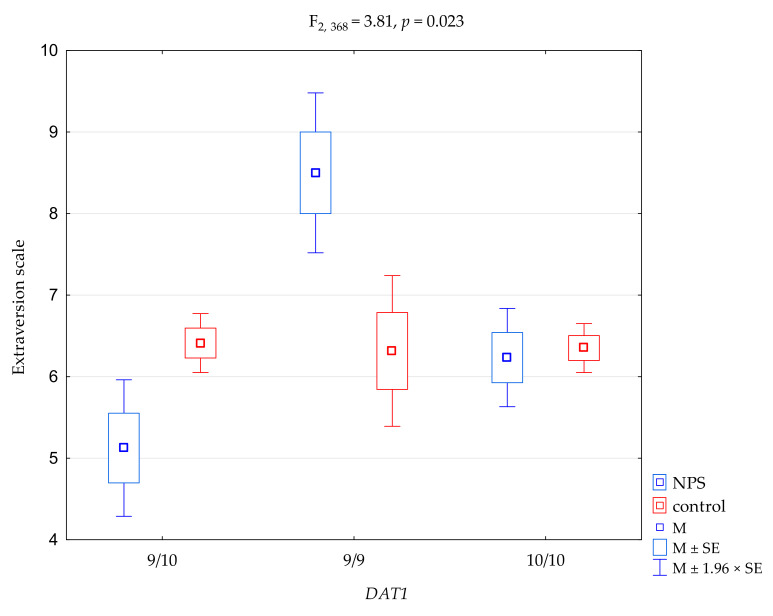
Interaction between users of ‘new psychoactive substances’ (NPS)/controls, *DAT1* polymorphisms, and the NEO-FFI extraversion scale. M, mean; SE, standard error.

The results of the 2 × 3 factorial ANOVA of the NEO Five-Factor Personality Inventory (NEO-FFI) and the State-Trait Anxiety Inventory (STAI) sten scales, and the scale of NEO-FFI, are summarized in Table 4. We found a significant result when comparing the NEO-FFI extraversion scale and *DAT1* polymorphisms (F_2, 368_ = 3.24, *p* = 0.040), accounting for 1.7% of the variance. For the interactions, we found a significant result when comparing the groups (NPS users vs. controls) for the NEO-FFI neuroticism scale and *DAT1* polymorphisms (Figure 1, F_2, 368_ = 4.23, *p* = 0,015), accounting for 2.2% of the variance. The results of the post hoc test are included in Table 5. For the interactions, we found a significant result when comparing the groups (NPS users vs. controls) for the NEO-FFI extraversion scale and *DAT1* polymorphisms (Figure 2, F_2, 368_ = 3.81, *p* = 0.023), accounting for 2.0% of the variance. Post hoc analysis is shown in Table 4. The results of the post hoc test are included in Table 5.

## 4. Discussion

Compared to the controls, we found no statistically significant differences in the genotype frequency between *DAT1* polymorphism variants, in the NPS users. This result may arise because new psychoactive substances are used by different groups, including people who take drugs recreationally, and because NPS users are a very heterogeneous group in terms of the substances taken. Designer drugs interact pharmacologically with various monoaminergic targets. Stimulants such as pipradrols and pyrovalerone cathinones inhibit the transport of dopamine; noradrenaline, amphetamines, and methamphetamine-like cathinones induce the release of these monoamines; entactogens such as phenylpiperazines, aminoindanes, and MDMA-like cathinones can enhance serotonin hallucin release; and in turn, ecstasy, tryptamines, and hallucinogenic phenethylamines are direct agonists at serotonergic 5-HT2A receptors [35]. A similar result was obtained in studies conducted on more homogeneous populations. In a study by Tzeng and colleagues [32] on a large cohort of Chinese male subjects diagnosed with amphetamine use disorder avoidance, they did not find an association between the *DAT1* gene and amphetamine dependence.

The main findings of our study are that interactions between *DAT1* polymorphism, neuroticism, and NPS use were found. Neuroticism is a personality dimension that involves emotional reactivity [36,37,38,39]. Individuals whose scores are high for neuroticism are more sensitive to negative mood states and have excessive vulnerability to environmental stressors. Neuroticism is thought to be genetically determined, with an estimated heritability of 40 to 60%, and may be involved in the susceptibility, onset, and course of mental disorders, including substance use disorder. In the existing literature, we found no papers which focused on analysis of personality dimensions of people who use various NPS, which are different from synthetic cannabinoids, meaning we can only compare our results with the results of research conducted on people with a diagnosis of SUD, which differs from NPS or synthetic cannabinoid use disorder. In a previous study [31], we showed that in the examined group of patients diagnosed with substance use disorder, which is different to NPS use disorder, no main effects of *DAT1* polymorphisms were found for any personality dimensions assessed using the NEO-FFI, but the main effects of *DAT1* polymorphisms approximated to the statistical significance for the agreeableness scale. Meta-analyses disclosed that high neuroticism and low conscientiousness were linked with substance use disorders [40]. Terracciano et al. [8] showed that low scores of conscientiousness, and high scores of neuroticism, were associated with the use of drugs. In this research, no correlation between the personality traits of extraversion, openness, conscientiousness, and agreeableness and *DAT1* polymorphism was found. In the group of NPS users, higher levels of neuroticism were found compared to the control group, whereas scores for extraversion and agreeableness were lower. People with low scores for agreeableness tend to be distant, unfriendly, and uncooperative, whereas people with low scores on extraversion are introverted. Introverted people are less involved in social activities; they are quiet and keep to themselves.

Analysis of the *DAT1* gene and the personality traits measured using the TPQ test was also performed by Tzeng and colleagues [36] on a large cohort of Chinese male subjects diagnosed with amphetamine use disorder. Novelty seeking and harm avoidance scores were higher in the case group than in the controls, but the analyzed polymorphism did not influence the scores. The explanation of these differences can be found in ethnic differences; the minor allele frequencies of the promoter and the 3′ VNTR polymorphisms are lower in Asians than in European Caucasian and Brazilian populations [36,41,42,43,44]. Kazantseva et al. [45] suggested that the 3′ VNTR and rs27072 of *DAT1* were associated with personality trait persistence in healthy Caucasian individuals.

A survey of the personality profile in alcohol, nicotine, cannabis, and gambling disorder in 3785 twins and siblings from the Australian Twin Registry showed that high neuroticism, low agreeableness, and low conscientiousness were associated with all four addictive disorders. In this research, neuroticism, agreeableness, and conscientiousness were associated with the general propensity for developing an addictive disorder and may, in part, explain their co-occurrence. However, they may be more broadly associated with the propensity for any psychiatric disorder [46]. Similarly, in our results, SUD was associated in the existing research with neuroticism and was also significantly associated with low extraversion and low agreeableness [40]. Compared to the control group, a higher severity of anxiety symptoms—both in terms of trait and state—was found in the group of NPS users. Numerous previous studies confirmed that personality features—anxiety and emotional distress—are associated with an early onset of drug use [47]. Neuroticism and anxiety were reported as correlating most strongly with coping motives for substance use [48]. It was also confirmed that females are more prone to using drugs in order to cope with negative emotions, such as anxiety [49,50]. Low anxiety sensitivity predicted increased motives for alcohol use, whereas high anxiety sensitivity predicted adjustment motives for alcohol and cannabis use, and high trait anxiety predicted coping motives for alcohol and cigarette use [51].

## 5. Conclusions

Conclusively, in this research, statistically significant interactions between neuroticism, the use of new psychoactive substances, and *DAT1* polymorphism were found. NPS users with 9/10 genotype variants had the highest levels of neuroticism compared to the control group. These associations reveal that psychological factors combined with genetic data enable a better understanding of the pathogenesis of addiction. The results may support the idea that neuroticism and anxiety correlate strongly with coping motives for using NPS.

## Figures and Tables

**Table 1:** Hardy–Weinberg law for the NPS users and control subjects.

Hardy–Weinberg Equilibrium Calculator Including Analysis for Ascertainment Bias		Observed (Expected)		Test χ^2^	
				χ^2^	*p*-Value
*DAT1* NPS users	9/9	2 (2.68)	9 allele freq = 0.19 10 allele freq = 0.81	0.268	>0.05
	9/10	24 (22.63)			
	10/10	47 (47.68)			
*DAT1* control subjects	9/9	19 (19.19)	9 allele freq = 0.25 10 allele freq = 0.75	0.003	>0.05
	9/10	114 (113.62)			
	10/10	168 (168.19)			

*p*—statistical significance in χ^2^ test.

**Table 2:** Frequency of genotypes of the *DAT1* gene polymorphisms in the NPS users and controls.

Group	*DAT1* Genotype Alleles				
	9/9 *n* (%)	9/10 *n* (%)	10/10 *n* (%)	9 *n* (%)	10 *n* (%)
NPS users	2	24	47	28	118
*n* = 73	(0.03)	(0.33)	(0.65)	(0.19)	(0.81)
Control	19	114	168	152	450
*n* = 301	(0.06)	(0.39)	(0.56)	(0.25)	(0.75)
χ^2^	2.48			2.37	
*p*-value	0.289			0.124	

*p*—statistical significance in χ^2^ test; N—number of subjects.

**Table 3:** STAI and NEO Five-Factor Inventory results (sten scale), shown as mean ± standard deviation for the healthy controls and the NPS users; significance of the difference was assessed by the Mann–Whitney U test.

STAI/NEO Five-Factor Inventory/	NPS Users (*n* = 73)	Control (*n* = 301)	Z	*p*-Value
STAI state/scale	6.08 ± 2.23	4.69 ± 2.14	4.614	0.0000 *
STAI trait/scale	7.34 ± 2.21	5.16 ± 2.18	6.889	0.0000 *
Neuroticism/scale	6.92 ± 2.39	4.67 ± 2.01	7.160	0.0000 *
Extraversion/scale	5.78 ± 2.16	6.37 ± 1.98	−2.113	0.0345 *
Openness/scale	4.84 ± 1.95	4.53 ± 1.61	1.248	0.2118
Agreeableness/scale	4.67 ± 2.06	5.60 ± 2.09	−3.446	0.0005 *
Conscientiousness/scale	5.82 ± 2.26	6.08 ± 2.15	−0.747	0.4550

*p*—statistical significance, Mann–Whitney U test; N—number of subjects, M ± SD, mean ± standard deviation. * Significant differences between groups.

**Table 4:** Differences in *DAT1* and the NEO Five-Factor Inventory scale or the STAI scale, between healthy control subjects and NPS users.

STAI/NEO Five-Factor Inventory			*DAT1*			ANOVA			
	NPS Users (*n* = 73)	Control (*n* = 301)	9/9 (*n* = 21)	9/10 (*n* = 138)	10/10 (*n* = 215)	Factor	F (*p*-Value)	ɳ^2^	Power (alfa = 0.05)
STAI state/scale	6.08 ± 2.23	4.69 ± 2.14	4.38 ± 2.25	5.05 ± 2.21	4.96 ± 2.24	intercept	F_1, 368_ = 341.56 (*p* < 0.0001 *)	0.481	1.000
						NPS users/control	F_1, 368_ = 6.30 (*p* = 0.012 *)	0.017	0.707
						*DAT1*	F_2, 368_ = 1.32 (*p* = 0.267)	0.007	0.285
						NPS users/control × *DAT1*	F_2, 368_ = 1.23 (*p* = 0.291)	0.007	0.269
STAI trait/scale	7.34 ± 2.21	5.16 ± 2.18	5.00 ± 2.07	5.74 ± 2.36	5.54 ± 2.36	intercept	F_1, 368_ = 457,68 (*p* < 0.0001 *)	0.554	1.000
						NPS users/control	F_1, 368_ = 15.07 (*p* = 0.0001 *)	0.039	0.972
						*DAT1*	F_2, 368_ = 1.22 (*p* = 0.295)	0.007	0.266
						NPS users/control × *DAT1*	F2,368 = 0.39 (*p* = 0.673)	0.002	0.114
Neuroticism/scale	6.92 ± 2.39	4.67 ± 2.01	4.48 ± 2.11	5.12 ± 2.36	5.16 ± 2.22	intercept	F_1, 368_ = 415.23 (*p* < 0.0001 *)	0.530	1.000
						NPS users/control	F_1, 368_ = 14.02 (*p* = 0.0002 *)	0.037	0.962
						*DAT1*	F_2,36 8_ = 2.62 (*p* = 0.073)	0.014	0.521
						NPS users/control × *DAT1*	F_2, 368_ = 4.23 (*p* = 0.015 *)	0.022	0.739
Extraversion/scale	5.78 ± 2.16	6.37 ± 1.98	6.52 ± 2.06	6.19 ± 2.04	6.33 ± 2.01	intercept	F_1, 368_ = 599.92 (*p* < 0.0001 *)	0.620	1.000
						NPS users/control	F_1, 368_ = 0.240 (*p* = 0.624)	0.001	0.078
						*DAT1*	F_2, 368_ = 3.24 (*p* = 0.040 *)	0.017	0.615
						NPS users/control × *DAT1*	F_2, 368_ = 3.81 (*p* = 0.023 *)	0.020	0.691
Openness/scale	4.84 ± 1.95	4.53 ± 1.61	4.29 ± 1.52	4.49 ± 1.78	4.68 ± 1.63	intercept	F_1, 368_ = 409.43 (*p* < 0.0001 *)	0.527	1.000
						NPS users/control	F_1, 368_ = 0.055 (*p* = 0.814)	0.0001	0.056
						*DAT1*	F_2, 368_ = 0.57 (*p* = 0.564)	0.003	0.145
						NPS users/control × *DAT1*	F_2, 368_ = 0.14 (*p* = 0.872)	0.001	0.071
Agreeableness/scale	4.67 ± 2.06	5.60 ± 2.09	4.81 ± 1.63	5.30 ± 2.18	5.55 ± 2.10	intercept	F_1, 368_ = 356,66 (*p* < 0.0001 *)	0.492	1.000
						NPS users/control	F_1, 368_ = 0.24 (*p* = 0.623)	0.001	0.078
						*DAT1*	F_2, 368_ = 0.68 (*p* = 0.507)	0.004	0.164
						NPS users/control × *DAT1*	F_2, 368_ = 1.15 (*p* = 0.314)	0.006	0.254
Conscientiousness/scale	5.82 ± 2.26	6.08 ± 2.15	6.62 ± 2.33	6.04 ± 2.10	5.96 ± 2.20	intercept	F_1, 368_ = 452.91 (*p* < 0.0001 *)	0.552	1.000
						NPS users/control	F_1, 368_ = 0.20 (*p* = 0.654)	0.0005	0.073
						*DAT1*	F_2, 368_ = 0.38 (*p* = 0.686)	0.002	0.111
						NPS users/control × *DAT1*	F_2, 368_ = 0.44 (*p* = 0.638)	0.002	0.123

* Significant differences between groups.

**Table 5:** Post hoc analysis of interactions between users of ‘new psychoactive substances’ (NPS)/controls, *DAT1* polymorphisms, and the NEO-FFI neuroticism/extraversion scale.

*DAT1* and NEO-FFI Neuroticism Scale						
	{1} M = 7.87	{2} M = 5.50	{3} M = 6.49	{4} M = 4.54	{5} M = 4.37	{6} M = 4.78
NPS *DAT1* 9/10 {1}		0.1207	0.0081 *	0.0000 *	0.0000 *	0.0000 *
NPS *DAT1* 9/9 {2}			0.5093	0.5186	0.4636	0.6286
NPS *DAT1* 10/10 {3}				0.0000 *	0.0002 *	0.0000 *
control *DAT1* 9/10 {4}					0.7331	0.3373
control *DAT1* 9/9 {5}						0.4065
control *DAT1* 10/10 {6}						
***DAT1* and NEO-FFI Extraversion Scale**						
	{1} M = 5.12	{2} M = 8.50	{3} M = 6.23	{4} M = 6.41	{5} M = 6.32	{6} M = 6.35
NPS *DAT1* 9/10 {1}		0.0226	0.0279	0.0044	0.0536	0.0053
NPS *DAT1* 9/9 {2}			0.1179	0.1447	0.1431	0.1322
NPS *DAT1* 10/10 {3}				0.6079	0.8807	0.7231
control *DAT1* 9/10 {4}					0.8459	0.8016
control *DAT1* 9/9 {5}						0.9418
control *DAT1* 10/10 {6}						

* Significant statistical differences; M—mean; {1–6}—the various polymorphisms of the DAT1 gene in NPS and control.

## Data Availability

Not applicable.
